# A case report about focal status epilepticus as first presentation in Alzheimer’s disease: finding the culprit

**DOI:** 10.1186/s12883-024-03979-4

**Published:** 2024-12-18

**Authors:** Astrid Devulder, Greet Vanderlinden, Evy Cleeren, Valerie Goovaerts, Tom Theys, Koen Van Laere, Wim Van Paesschen

**Affiliations:** 1 Laboratory for Epilepsy Research, KU Leuven, Belgium; 2 Department of Neurology, University Hospitals Leuven, Herestraat 49, Leuven, 3000 Belgium; 3 Nuclear Medicine and Molecular Imaging, Department of Imaging and Pathology, KU Leuven, Belgium; 4 Division of Nuclear Medicine, University Hospitals Leuven, Leuven, Belgium; 5 Research Group Experimental Neurosurgery and Neuroanatomy, KU Leuven, Belgium; 6 Department of Neurosurgery, University Hospitals Leuven, Leuven, Belgium

**Keywords:** Alzheimer’s disease, Case report, Hippocampal atrophy, Tau pathology, Temporal lobe epilepsy

## Abstract

**Background:**

Neuronal hyperexcitability has been proposed to play a key role in Alzheimer’s disease (AD). Understanding the relation between this enhanced excitability and AD pathology could provide a window for therapeutic interventions. However epileptiform activity is often subclinical, hidden on scalp EEG and very challenging to assess with current diagnostic modalities.

**Case presentation:**

A woman in her sixties presented with acute confusion. Despite a normal scalp electroencephalogram (EEG), magnetic resonance imaging (MRI) showed cytotoxic edema of the right mesial temporal lobe and hippocampal hypermetabolism was present on ([^18^F]-fluoro-2-deoxyglucose positron emission tomography (PET). Bilateral foramen ovale (FO) electrodes were placed to directly record mesial temporal activity and revealed continuous mesial temporal epileptic activity, while scalp EEG remained normal. After recovery, a new diagnosis of AD was established on cerebrospinal fluid. The lateralization of the epileptiform activity was congruent with the predominant side of tau pathology in the mesial temporal cortex on ^18^F-MK6240 PET. On follow-up MRI, two and five months later, the right hippocampus became atrophic.

**Conclusion:**

This case highlights the significant role of neuronal hyperexcitability in early AD pathogenesis and how shared mechanisms between AD and epilepsy can complicate clinical management.

**Supplementary Information:**

The online version contains supplementary material available at 10.1186/s12883-024-03979-4.

## Background

A pathological hallmark in Alzheimer’s disease (AD) is the deposition of misfolded hyperphosphorylated tau as neurofibrillary tangles [1], of which the spatial distribution strongly correlates with clinical symptoms [2]. Evidence from both mice and human studies suggests a role for neuronal activity in the release and the trans-neuronal and trans-synaptic spread of tau [3–5], which follows a pattern of entorhinal functional connectivity [6]. It has been proposed that hippocampal activity follows an inverted U shape pattern: hyperactivity early in the disease course followed by hypoactivity in later disease stages [7]. Increased neuronal activity in AD can manifest as epileptiform discharges and subclinical seizures and is associated with faster cognitive decline [8], while reducing hyperexcitability can have beneficial effects on cognitive functions [9]. In the investigation of potential treatment targets, finding biomarkers is essential. However, early detection of subclinical epileptiform activity remains a challenge [10]. In a patient who presented with acute confusion, we directly measured hyperexcitability from ictal [^18^F]-fluoro-2-deoxyglucose (^18^F-FDG) positron emission tomography (PET) and recorded epileptic activity from intracranial electrodes which could not be obtained from surface electroencephalograms (EEG). To better understand the interplay between network hyperexcitability and AD pathogenesis we quantified tau in vivo using ^18^F-MK6240 PET.

## Case presentation

**Fig. 1:**
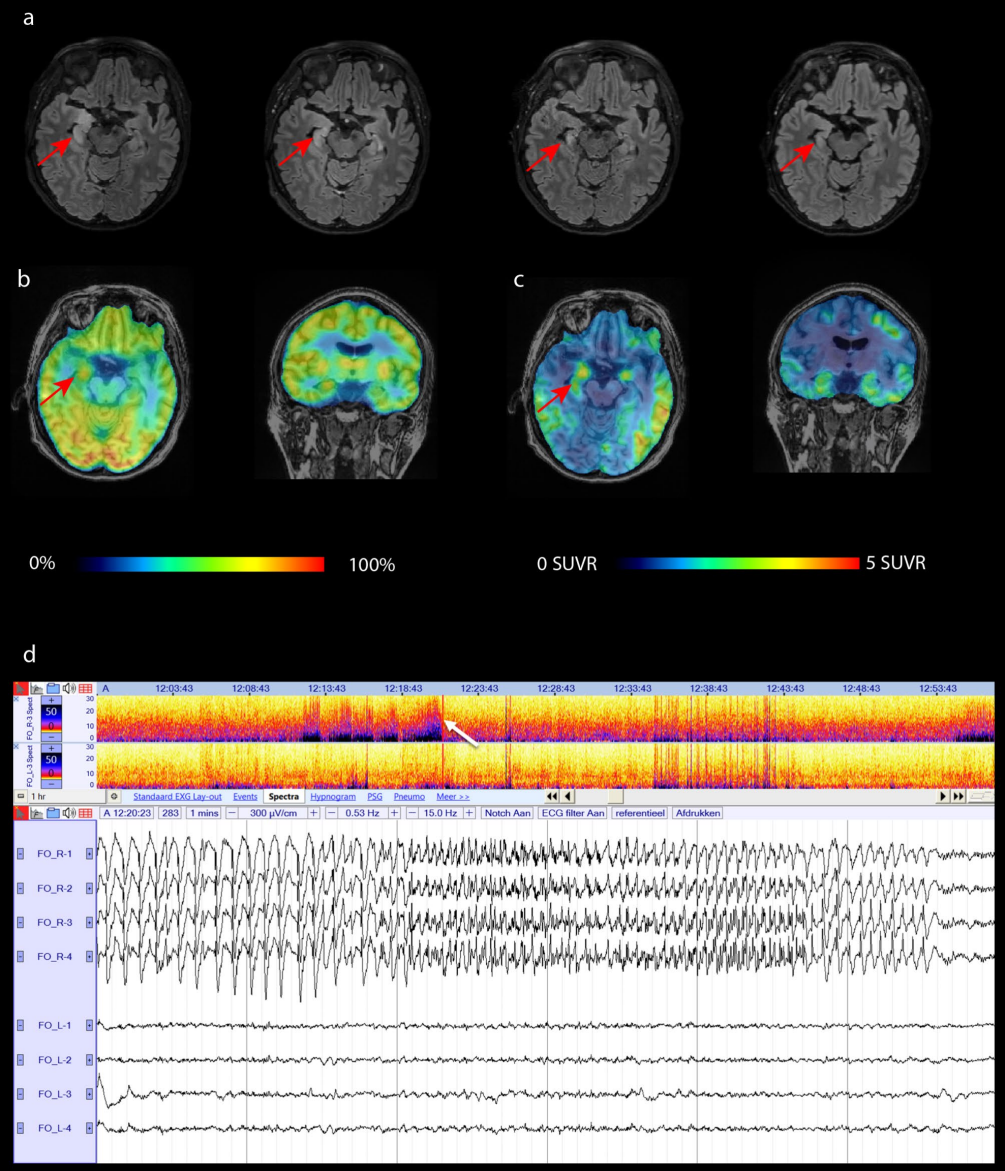
Imaging and EEG data. (**A**): Sequential brain MRI: axial FLAIR images. From left to right: Cytotoxic edema of the right hippocampus and amygdala (arrow) at presentation (MTA right 0/1); Decreased edema of the right hippocampus and amygdala (arrow) 20 days later (MTA right 1); Atrophy of the right hippocampus (arrow) with increased T2/FLAIR signal consistent with hippocampal sclerosis two months later (MTA right 2); Stable atrophy of right hippocampus with hippocampal sclerosis (arrow) five months later (MTA right 2). (**B**): Axial and coronal images of ^18^F-FDG PET at presentation with hypermetabolism of the right hippocampus (arrow). (**C**): Axial and coronal image of ^18^F-MK6240 tau PET one month later shows increased SUVR in the hippocampus (R > L) (arrow) and typical Braak neocortical brain regions, a pattern consistent with AD. (**D**): Intracranial EEG (bottom panel) obtained from FO electrodes and spectrogram (top panel) on day 2. Subclinical electrographic mesial temporal lobe seizure on right FO electrode (under treatment with levetiracetam): LPDs on right FO electrode with evolution in frequency and morphology and abrupt end. Spectrogram reveals first increased delta activity (LPDs) with evolution to faster activity on the right FO electrode at the time of seizure onset (white arrow). Continuous EEG of 60 s. A referential montage is shown where the upper four channels represent the four contact points (from anterior to posterior) of the right FO electrode, and the lower four channels represent the left FO electrode. Filters at 0.53–15.0 Hz, 300µV/cm. The top panel represents a one hour spectrogram where the upper spectrogram corresponds to the third contact of the right FO electrode, and the lower spectrogram to the third contact of the left FO electrode

A woman in her sixties, right-handed, with no relevant medical history reported mild memory complaints over the last year, e.g. remembering names and appointments, losing personal items, difficulty remembering recent conversations. There was no family history of dementia. She was admitted to the emergency department after her neighbor heard a fall and had witnessed a confused state one hour earlier that day. On clinical neurological examination there was fluctuating disorientation, strange affect and an amnestic deficit, without typical signs of a seizure such as tongue bite, incontinence, gaze deviation, automatisms or rhythmic movements. Systemic and brain infections, auto-immune encephalitis, intoxication, metabolic causes, and acute stroke were excluded by blood, urine, cerebrospinal fluid (CSF) analysis and computed tomography angiography. Seizure activity with an epileptic fall was our working hypothesis, for which a continuous 48-hour video-scalp-EEG was performed. Slower activity, consisting of intermittent theta and delta activity with an alternating lateralization over both hemispheres, was seen but no epileptiform activity was recorded. Brain magnetic resonance imaging (MRI) showed cytotoxic edema of the right hippocampus and amygdala (Fig. 1a) and anti-seizure medication (ASM) (levetiracetam 1000 mg twice daily) was initiated. ^18^F-Fluoro-ethyl-tyrosine PET, to exclude a brain tumor, was normal. Her confusion improved, but the amnestic deficit and fluctuating disorientation persisted, with deficits in orientation, delayed recall and complex task on neuropsychological testing. ^18^F-FDG PET, performed under levetiracetam treatment, showed a hypermetabolic focus in the right hippocampus (Fig. 1b), consistent with our clinical suspicion of focal non-convulsive status epilepticus [11]. Mild left frontotemporoparietal and bilateral midline parietal hypometabolism were also present, and to confirm a diagnosis of focal status epilepticus, bilateral foramen ovale (FO) electrodes were implanted to measure mesial temporal lobe activity during five days. An electrographic seizure on the right FO electrode during wakefulness (Fig. 1d), and abundant (i.e., around 50% of the recording) right lateralized periodic discharges (LPDs) of intermediate duration (i.e., up to 10 min) with an average frequency of 1 Hz (0.5 – 2 Hz) were observed. After levetiracetam was increased to 1500 mg twice a day and lacosamide 300 mg twice daily was associated, no more seizures were recorded and spike frequency decreased with 40% from 442 spikes/h to 259 spikes/h, although LPDs remained frequent. Her confusional state disappeared and she became oriented in time and space with a residual amnestic deficit. On neuropsychological assessment two weeks later, deficits in verbal memory, working memory and complex attention were present, indicative of amnestic mild cognitive impairment (MCI) (Additional file 1). An AD diagnosis was confirmed on CSF analysis (increased total tau of > 1300 pg/mL (normal < 545 pg/mL) influenced by acute neuronal damage, increased phospho-tau of 84 pg/mL (normal < 75 pg/mL), Aβ 1–42 of 328 pg/mL, Aβ 1–40 of 5585 pg/mL and a decreased Aβ 1–42/1–40 ratio of 0.059 (normal > 0.096) [18]. F-MK-6240 tau-PET showed an increased tracer binding (also quantified by standard uptake value with reference to cerebellum, SUVR) in the mesial temporal brain regions predominantly on the right side as well as in the cingulate gyrus and typical Braak neocortical brain regions predominantly on the left side (Fig. 1c). The asymmetry index (AI) for the ^18^F-MK-6240 tracer uptake in the mesial temporal brain regions was calculated as 200 * (right – left) / (right + left) [2]. This index can be interpreted as an asymmetric tracer binding, and showed lateralization towards the epileptic hemisphere (AI mesial temporal brain region = 16%). At discharge from hospitalization donepezil 5 mg was started and her ASM consisted of levetiracetam 1500 mg twice daily and lacosamide 200 mg twice daily. Follow-up MRIs, respectively two and five months later, showed decreased edema in the right hippocampus and amygdala evolving to hippocampal atrophy with accompanying gliosis (mesial temporal atrophy score, MTA 2) (Fig. 1a) [12]. On clinical examination after six months the patient mentioned difficulties with short term memory and instrumental activities of daily living. Neuropsychological testing revealed remaining mild shortcomings on delayed recall, complex task and repetition. Technical details are available in Additional file 2.

## Discussion

This report highlights the challenge clinicians face to diagnose mesial temporal lobe seizures in patients with diagnosed, and even more so undiagnosed AD. Scalp EEG has limited sensitivity in detecting mesial temporal lobe epileptiform activity in such cases [10, 13]. In situations with a high clinical suspicion of seizures such as cognitive fluctuations [14, 15] or cytotoxic edema on MRI, other modalities such as ^18^F-FDG PET and invasive recordings can be warranted to detect ‘hidden’ seizures [10, 11]. These studies are not available in most hospitals and ask for a cooperative patient, which can be challenging in AD. In this patient, who presented with acute confusion, we directly measured hyperexcitability from ictal ^18^F-FDG PET and recorded epileptic activity from FO electrodes which could not be obtained from surface EEG.

A combined FO-scalp EEG study also provides a unique opportunity to monitor the effect of ASM on epileptiform activity. Under standard anti-seizure treatment, clinically silent seizures can be recorded and ASM can be adjusted accordingly. We demonstrate that neuronal hyperexcitability during early AD course is difficult to detect and might continue despite ASM with deleterious consequences on cognitive functioning [8]. This might explain the modest effect of treatment with ASM on cognitive functions in AD patients with subclinical epileptiform activity on scalp EEG [9]. As neuronal hyperexcitability is a potential treatment target in AD, it is essential to find a reliable biomarker to detect and monitor epileptiform activity in the AD population.

AD was thought to be a symmetric disease, however with the implementation of tau PET, asymmetry in tau pathology has been observed [16–18]. Global hemispheric asymmetric tau distribution was associated with a higher tau pathological burden, earlier age of onset and faster cognitive decline [18]. The underlying mechanisms for the lateralization in tau distribution are not well investigated, however epileptiform activity might play a role [3–5]. Congruent lateralization between epileptic activity and asymmetry in tau pathology in AD has been observed [13, 19]. We documented an evolution from cytotoxic edema during ictal hippocampal activity to hippocampal atrophy with sclerosis, an abnormality which is common in both AD and drug-resistant mesial temporal lobe epilepsy. In a histopathological study of drug-resistant epilepsy with hippocampal sclerosis abnormal hyperphosphorylated tau was present in 94% of cases [20]. Despite the limitations, that no amyloid-ß PET was available, and the lack of longitudinal biomarker measurements on CSF or PET, this case provides in vivo evidence to support the hypothesis that an association exists between neuronal activity and tau pathology in early AD pathogenesis [21, 22], however no causal direction can be established.

## Electronic supplementary material

Below is the link to the electronic supplementary material.

Supplementary Material 1: Additional file 1: Word document (.docx). Neuropsychological assessment. Extensive neuropsychological testing

Supplementary Material 2: Additional file 2: Word document (.docx). Methods. Description of the methods used to analyse the data

## Data Availability

The data is available upon reasonable request.

## Abbreviations

Aβ: Amyloid beta
AD: Alzheimer’s disease
AI: Asymmetry index
ASM: Anti-seizure medication
CSF: Cerebrospinal fluid
EEG: Electroencephalogram
FO: Foramen ovale
LPDs: Lateralized periodic discharges
MCI: Mild cognitive impairment
MRI: Magnetic resonance imaging
MTA: Mesial temporal atrophy score
PET: Positron emission tomography
^18^F-FDG: [^18^F]-fluoro-2-deoxyglucose
