# Supplementary material for: A case report about focal status epilepticus as first presentation in Alzheimer’s disease: finding the culprit
Source: BMC Neurol. 2024 Dec 18;24:478. doi: 10.1186/s12883-024-03979-4 (PMC11654376; doi:10.1186/s12883-024-03979-4)
Supplement: Supplementary file 1 — Supplementary Material 1: Additional file 1: Word document (.docx). Neuropsychological assessment. Extensive neuropsychological testing [file 12883_2024_3979_MOESM1_ESM.docx]

**ADDITIONAL FILE 1: NEUROPSYCHOLOGICAL ASSESSMENT (27-03-2023)**

A.1 MEMORY

A.2 ATTENTION & EXECUTIVE FUNCTIONS

A.3 LANGUAGE

A.4 VISUAL PERCEPTION AND CONSTRUCTIONAL PRAXIS

A.5 MOOD

A.1 MEMORY

* Logical Memory (COTESS)

-textual elements 9.5/23 Pc 50-84

-context elements 3/5 Pc 16

* Rey Auditory Verbal Learning Test (RAVLT)

-trial 1 5/15 Pc 14

-trial 2 6/15

-trial 3 9/15

-trial 4 9/15

-trial 5 9/15 Pc 4

-total (1-5) 38/75 Pc 3

-number of learned items 4

-repetitions 2

-intrusions 2 (dog, goose)

-delayed recall (DR) 7/15 Pc 1

-retention (A7/A5) 78%

-delayed recognition 10/15

-correct 13/15 Pc 20-30

-false positive 3 Pc 10

* Rey Visual Design Learning Test (RVDLT)

-trial 1 3.5/15 Pc 45

-trial 2 6/15

-trial 3 5.5/15

-trial 4 6.5/15

-trial 5 5/15 Pc 20

-total (1-5) 26.5/75 Pc 43

-number of learned items 3

-repetitions 0

-intrusions 17

-delayed recall (DR) 4.5/15 Pc 22

-retention 90%

-delayed recognition 9/15

-correct 13/15 Pc 70-80

-false positives 4 Pc 30

* Visual Association Test (VAT): short version

-trial 1 6/6 Pc >29

A.2 ATTENTION & EXECUTIVE FUNCTIONS

* Digit Span

-forward (number of series) 5

-forward (longest streak) 5 Z = -1

-backward (number of series) 4

-backward (longest streak) 3 Z = -2

* Stroop Color Word Test (SCWT)

-word reading 52" Z = 1.4

-color naming 63" Z = 1.2

-interference 323" Z = 17

-interference factor 265.5 Z = 20

-false 16 Z = 16

-autocorrections 9

* Letter Digit Substition Test (LDST)

-correct 30 Z = 0

* Trail Making Test (TMT)

-part A 33" Z = 0

-part B 113" Z = 2

-B/A 3.4 Pc 5

*Fluency

-semantic: animals 24 Z = 1.1

-phonemic 63 Z = 2.6

P (rep: 4, fal: 0) 19 Z = 0.9

N (rep: 0, fal: 1) 18

T (rep: 4, fal: 0) 24

* Behavioral Assessment of Dysexecutive Syndrome (BADS)

-key search test 1 Pc 11

A.3 LANGUAGE

* Boston Naming Test (BNT)

-correct 54/60 Pc 63

A.4 VISUAL PERCEPTION AND CONSTRUCTIONAL PRAXIS

* Visual Object & Space Perception (VOSP)

-cube analysis 10/10 Pc 77

* Constructional Praxis (ADAS-COG)

-overlapping rectangles 1/1

-cube 1/1

A.5. MOOD

* Beck Depression Inventory (BDI-II)

-total 9/63 minimal

* Depression, Anxiety and Stress Scale (DASS-42)

-depression 16/42 moderate

-anxiety 9/42 mild

-stress 23/42 moderate

Abbreviations: RS = raw score; SS = scaled score; Pc= percentile
