# Supplementary material for: A case report about focal status epilepticus as first presentation in Alzheimer’s disease: finding the culprit
Source: BMC Neurol. 2024 Dec 18;24:478. doi: 10.1186/s12883-024-03979-4 (PMC11654376; doi:10.1186/s12883-024-03979-4)
Supplement: Supplementary file 2 — Supplementary Material 2: Additional file 2: Word document (.docx). Methods. Description of the methods used to analyse the data [file 12883_2024_3979_MOESM2_ESM.docx]

**ADDITIONAL FILE 2: METHODS**

A.1 Foramen ovale (FO) electrode placement

After explaining the risks and benefits, informed consent for the placement of FO electrodes was obtained. The placement of bilateral FO electrodes (AdTech, US) was performed in the operating room under general anesthesia (dorsal decubitus, endotracheal tube). A post-op CT scan was performed to confirm correct positioning and exclude intracranial hemorrhage.

A.2 Scalp EEG and FO electrode recordings

Scalp electrodes were placed according to the International 10-20 system with additional lower temporal line electrodes (F10, T10, P10, F9, T9, P9).^1^ Recordings were obtained with BrainRT software (OSG BVBA, Kontich, Belgium) with a sampling rate of 256 Hz. Scalp EEG and FO recordings were visually analyzed according to International Federation for Clinical Neurophysiology criteria.^2,3^

A.3 Cognitive testing

Neuropsychological examination was performed by an experienced neuropsychologist (V.G.).

A.4 Laboratory testing

CSF amyloid and tau analysis were performed at the University Hospitals Leuven.

A.5 Imaging

MRI brain images were obtained with and without gadolinium contrast on a 3T Philips MRI, with following sequences: axial DWI, axial SWI, axial T1, axial T2, axial FLAIR, coronal T1, coronal FLAIR, sagittal T1, sagittal FLAIR.

^18^F-FDG PET images were obtained on a Siemens HiRez PET/CT with static acquisition 30 to 45 minutes after 172 MBq ^18^F-FDG injection under EEG monitoring.

^18^F-FET PET image was obtained on a Siemens HiRez PET/CT with dynamic acquisition 0 to 40 minutes after 185 MBq tracer injection.

^18^F-MK-6240 PET/MR images were obtained on a simultaneous GE Signa PET/MR with static acquisition 90 to 120 minutes post tracer injection of 165 MBq.

All scans, except ^18^F-MK-6240 PET/MR, were performed for clinical purposes. Interpretation was done by experienced neuroradiologist or nuclear medicine physician.

A.6 Research ethics

For each investigation the rationale and risk/benefits were explained. Informed consent for publication was obtained by patient. The study was approved by the Ethical Committee of University Hospitals Leuven (clinical.trials.gov NCT03617497).

A.7 References

1. Seeck M, Koessler L, Bast T, Leijten F, Michel C, Baumgartner C, He B, Beniczky S. The standardized EEG electrode array of the IFCN. J Clin Neurophysiol 2017; 128: 2070-77.

2. Hirsch LJ, Fong MWK, Leitinger M, LaRoche SM, Beniczky S, Abend NS, Lee JW, Wusthoff CJ, Hahn CD, Westover MB, Gerard EE, Herman ST, Haider HA, Osman G, Rodriguez-Ruiz A, Maciel CB, Gilmore EJ, Fernandez A, Rosenthal ES, Claassen J, Husain AM, Yoo JY, So EL, Kaplan PW, Nuwer MR, van Putten M, Sutter R, Drislane FW, Trinka E, Gaspard N. American Clinical Neurophysiology Society's Standardized Critical Care EEG Terminology: 2021 Version. J Clin Neurophysiol 2021; 38: 1-29.

3. Kural MA, Duez L, Sejer Hansen V, Larsson PG, Rampp S, Schulz R, Tankisi H, Wennberg R, Bibby BM, Scherg M, Beniczky S. Criteria for defining interictal epileptiform discharges in EEG: A clinical validation study. Neurology 2020; 94: e2139-e47.
